# Supplementary material for: Opposite changes in APP processing and human Aβ levels in rats carrying either a protective or a pathogenic APP mutation
Source: eLife. 2020 Feb 5;9:e52612. doi: 10.7554/eLife.52612 (PMC7018507; doi:10.7554/eLife.52612)
Supplement: Supplementary file 1. [file elife-52612-supp1.docx]

**Supplementary file 1. Generation of App KI rats.**

Generation of *App^δ7^*, *App^s^*, and *App^h^* rats was as described previously (Tambini et al., 2019; 10.1111/acel.13033). We created a Long-Evans rat model with point mutations GCG>ACG, GGA>CGA, TTC>TAC, CGC>CAT at the rat *App* locus by CRISPR/Cas-mediated genome engineering. These mutations will create a rat that carries an *App* gene with the humanized Aβ sequence and the protective Icelandic A673T mutation:

Rat Aβ43: DAEFGHDSGFEVRHQKLVFFAEDVGSNKGAIIGLMVGGVVIAT

Human Aβ43: DTEFRHDSGYEVHHQKLVFFAEDVGSNKGAIIGLMVGGVVIAT

Rat *vs*. human-protective Aβ43 sequence. The nucleotide changes introduced in Exon 16 of rat *App* will generate rats producing human Aβ peptides (differences are in white and boxed in black) with the protective Icelandic mutation (in red boxed in yellow).

gRNA targeting vector and oligo donor (with targeting sequence, flanked by 120bp homologous sequences combined on both sides) were designed as follows.

gRNA1: CTCAGAAGTGAAGATGGATGCGG

gRNA2: CGAAGTCCGCCATCAAAAACTGG

The links for a detailed description of the gRNA vectors can be found in the following sites:

gRNA1: <http://www.vectorbuilder.com/design/report/733e7a50-b705-45cf-8f8c-8b8206e6f174>

gRNA2: <http://www.vectorbuilder.com/design/report/2a73df2c-1d5c-4e69-b43a-ee6cfaea80c6>

5’-GACTCAGGGTCTGGGTTGACAAACATCAAGACAGAAGAGATCTCAGAAGTGAAGATGGATGCGGAGTTCGGACATGAT

TCAGGCTTCGAAGTCCGCCATCAAAAACTGGTATGCAAAAGATAATCTGCCTCTCCCCACAACTGACTGGCCAGATAG-3’

Rat *App* exon 16 targeted sequence. The gRNA1 and gRNA2 sequences are boxed in yellow and green, respectively.

The nucleotides targeted for mutation are in red and underlined.

5’-GACTCAGGGTCTGGGTTGACAAACATCAAGACAGAAGAGATCTCAGAAGTGAAGATGGATACGGAGTTCCGACATGAT

TCAGGCTACGAAGTCCATCATCAAAAACTGGTATGCAAAAGATAATCTGCCTCTCCCCACAACTGACTGGCCAGATAG-3’

Oligo donor sequence, with the mutated nucleotide in red.

Cas9 mRNA, gRNA generated by *in vitro* transcription and oligo donor were co-injected into zygotes. To verify CRISPR-induced mutation the pups were genotyped by PCR using the following rat *App* specific forward (F) and reverse (R) primers: F-CTTTCTCCAGTCTGTTTGCTTGCG; R-GCCTGCTTCCGTGCTTCCTTT. The PCR products were cloned into TA vectors: 10 *App* positive plasmids were sequenced with primer F. This analysis showed that RatID#88 had three types of mutant alleles (WT= Wild type):

WT 5’-GACAGAAGAGATCTCAGAAGTGAAGATGGATGCGGAGTTCGGACATGATTCAGGCTTCGAAGTCCGCCATCAAAAACT-3’

ID#88 5’-GACAGAAGAGATCTCAGAAGTGAAGATGGATACGGAGTTCCGACATGATTCAGGCTACGAAGTCCATCATCAAAAACT-3’

**Humanized Protective allele**. Sequence of the allele carrying the humanizing (in red and boxed) and Protective (in blue and boxed) mutations compared to the sequence of rat *App*. The gRNAs sequences are underlined.

WT 5’-GACAGAAGAGATCTCAGAAGTGAAGATGGATGCGGAGTTCGGACATGATTCAGGCTTCGAAGTCCGCCATCAAAAACT-3’

ID#88 5’-GACAGAAGAGATCTCAGAAGTGAAGATGGATGCGGAGTTCGGACATGATTCAGGCTTCGAAGTCCGCCATCAAAAACT-3’

**δ25 allele**. Sequence of the allele carrying a 25bp deletion (in black) compared to the sequence of rat *App*. The gRNAs sequences are underlined.

WT 5’-GACAGAAGAGATCTCAGAAGTGAAGATGGATGCGGAGTTCGGACATGATTCAGGCTTCGAAGTCCGCCATCAAAAACT-3’

ID#88 5’-GACAGAAGAGATCTCAGAAGTGAAGATGGATGCGGAGTTCGGACATGATTCAGGCTTCGAAGTCCGCCATCAAAAACT-3’

**δ44 allele**. Sequence of the allele carrying a 44bp deletion (in black) compared to the sequence of rat *App*. The gRNAs sequences are underlined.

Thus, RatID#88 was identified as a positive chimeric founder (F0-*App^p^* rat).

To identify potential off-target sites for gRNA1 and gRNA2, RatID#88 (F0-*App^p^* rat) has been analyzed for mutations in these most likely off-target mutation sites. Three potential off-target sites have been identified for gRNA1: CTCAGAAGTGAAGATGGATGCGG (mismatched bases with the targeting sequence are in red.) These sites have been amplified by PCR and sequenced.

**Off-target site on** [**chr**](http://uswest.ensembl.org/Mus_musculus/Gene/Summary?db=core;g=ENSMUSG00000022667;tl=zWaN4XVMtlTpM4Bv-1540378-359099822) **20:** 5526008 ATAAGAAATGAAGATGGATGCAG 5526030

Targeting sequence: CTCAGAAGTGAAGATGGATGCGG

A 353bp products was generated by PCR with the following F and reverse (R) oligos:

F: GCTGGTCTCCTCCAGGTTAGTGGA; R: TAGTTGGGTACATAATGTTGTCCACCCA

The PCR product was sequenced using primer F. Sequencing results comparing WT rat and RatID#88 showed that RatID#88 (F0-*App^p^* rat) had no off-target mutations in this site.

WT 5’-GATCGGCACTGCTCAGGCTAATAAGAAATGAAGATGGATGCAGAGCCTGGCTCCTGTGCAAGCGGGTG-3’

ID#88 5’-GATCGGCACTGCTCAGGCTAATAAGAAATGAAGATGGATGCAGAGCCTGGCTCCTGTGCAAGCGGGTG-3’

**Off-target site on** [**chr**](http://uswest.ensembl.org/Mus_musculus/Gene/Summary?db=core;g=ENSMUSG00000022667;tl=zWaN4XVMtlTpM4Bv-1540378-359099822) **1:** 886326539 CTCAGATGTGGAGATGGATGAAG 886326561

Targeting sequence: CTCAGAAGTGAAGATGGATGCGG

A 529bp products was generated by PCR with the following F and reverse (R) oligos:

F: TTGACAAGCGGCCAGGAGTGA; R: GCTATTGCTTCCTGGCTGATTTGG

The PCR product was sequenced using primer F. Sequencing results comparing WT rat and RatID#88 showed that RatID#88 (F0-*App^p^* rat) had no off-target mutations in this site.

WT 5’-TCTTGTAAAGCATGGTGTAGCAGAGGGCTTCTCAGATGTGGAGATGGATGAAGAAGACATGGGCTGGTGT-3’

ID#88 5’-TCTTGTAAAGCATGGTGTAGCAGAGGGCTTCTCAGATGTGGAGATGGATGAAGAAGACATGGGCTGGTGT-3’

**Off-target site on** [**chr**](http://uswest.ensembl.org/Mus_musculus/Gene/Summary?db=core;g=ENSMUSG00000022667;tl=zWaN4XVMtlTpM4Bv-1540378-359099822) **12:** 14202718 CTCAGCCGTGAAGATGGATGAGG 14202740

Targeting sequence: CTCAGAAGTGAAGATGGATGCGG

A 356bp products was generated by PCR with the following F and reverse (R) oligos:

F: TTCAACACTGGGAGACAACAGATCG; R: CTTTGTGTGGAGTAGGTGTCTGTCCTC

The PCR product was sequenced using primer F. Sequencing results comparing WT rat and RatID#88 showed that RatID#88 (F0-*App^p^* rat) had no off-target mutations in this site.

WT 5’-CAGGCCACTGCCCTTACACGGCTTCTCAGCCGTGAAGATGGATGAGGCTGCTGTGATGGGACTT-3’

ID#88 5’-CAGGCCACTGCCCTTACACGGCTTCTCAGCCGTGAAGATGGATGAGGCTGCTGTGATGGGACTT-3’

Two potential off-target sites have been identified for gRNA2: CGAAGTCCGCCATCAAAAACTGG**.** (mismatched bases with the targeting sequence are in red.) These sites have been amplified by PCR and sequenced.

**Off-target site on** [**chr**](http://uswest.ensembl.org/Mus_musculus/Gene/Summary?db=core;g=ENSMUSG00000022667;tl=zWaN4XVMtlTpM4Bv-1540378-359099822) **2:** 260814277 CCAGGTGAGCCATCAAAAACCAG 260814299

Targeting sequence: CGAAGTCCGCCATCAAAAACTGG

A 437bp long products was generated by PCR with the following F and R oligos:

F: TGAGTGCAAGATACAAGGATAATCAAGGA; R: CCCAGTTCAAGTGGTCACAAGAATAAC

The PCR product was sequenced using primer F. Sequencing results comparing WT rat and RatID#88 showed that RatID#88 (F0-*App^p^* rat) had no off-target mutations in this site.

WT 5’-CCAAACCTCTGAAGGACTCAAAGCTCCACCAGGTGAGCCATCAAAAACCAGTTGAGAAAGC-3’

ID#88 5’-CCAAACCTCTGAAGGACTCAAAGCTCCACCAGGTGAGCCATCAAAAACCAGTTGAGAAAGC-3’

**Off-target site on** [**chr**](http://uswest.ensembl.org/Mus_musculus/Gene/Summary?db=core;g=ENSMUSG00000022667;tl=zWaN4XVMtlTpM4Bv-1540378-359099822) **X:** 83926631 AGCAATGCGCCATCAAAAACCAG 83926653

Targeting sequence: CGAAGTCCGCCATCAAAAACTGG

A 790bp long products was generated by PCR with the following F and R oligos:

F: CCCACCACAATTACTGGGGC; R: AAATCCATCTTCACATACGGCG

The PCR product was sequenced using primer R. Sequencing results comparing WT rat and RatID#88 showed that RatID#88 (F0-*App^p^* rat) had no off-target mutations in this site.

WT 5’-AAACAAGATTTTCTTACGTTGTGAGCAATGCGCCATCAAAAACCAGGAGATGAACAGAATTTGAGTCA-3’

ID#88 5’-AAACAAGATTTTCTTACGTTGTGAGCAATGCGCCATCAAAAACCAGGAGATGAACAGAATTTGAGTCA-3’
